# Internet addiction and poor quality of life are significantly associated with suicidal ideation of senior high school students in Chongqing, China

**DOI:** 10.7717/peerj.7357

**Published:** 2019-07-17

**Authors:** Wo Wang, Dong Dong Zhou, Ming Ai, Xiao Rong Chen, Zhen Lv, Yan Huang, Li Kuang

**Affiliations:** 1 Mental Health Center, University-Town Hospital of Chongqing Medical University, Chongqing, China; 2 Department of Psychiatry, The First Affiliated Hospital of Chongqing Medical University, Chongqing, China; 3 GCP office, Chongqing Traditional Chinese Medicine Hospital, Chongqing, China

**Keywords:** Suicide attempt, Suicide ideation, Internet addiction, Adolescent

## Abstract

**Background:**

Adolescence is a vulnerable period of life, and many mental health and behavioral problems emerge during this particular period, including depression, internet addiction (IA), and suicidal behaviors. Poor quality of life (QOL) and IA have been found to be significantly associated with suicidal ideation (SI) among Chinese university students, of whom most have been adults. Nevertheless, their associations with SI are rarely studied among Chinese adolescents. The objective of this study was to examine these relationships in a representative adolescent sample of Chinese senior high school students, who are experiencing an enormous transition from childhood to adulthood.

**Methods:**

By using multi-stage sampling, a total of 26,688 students were successfully recruited from 29 senior high schools of a very large City in western China, Chongqing municipality. In this cross-sectional online survey, students’ demographic and lifestyle characteristics were collected with a standardized questionnaire. The Young’s IA Test, the Chinese Six-item QOL questionnaire, and item 15 of the Symptom Checklist-90-R were used to measure IA, QOL, and SI, respectively.

**Results:**

The 1-month prevalence of SI was 11.5% among students of senior high schools in Chongqing, China. Students with SI had significantly higher QOL scores (17.3 ± 3.7 vs. 13.7 ± 3.8, *P* < 0.001) and higher prevalence of IA (49.6% vs. 25.6%, *P* < 0.001) than those without SI. After controlling for demographic, lifestyle, and clinical covariates, IA (odd ratio (OR) = 1.15, *P* = 0.003) and a high QOL score (OR = 1.09, *P* < 0.001) remained significantly associated with SI.

**Conclusion:**

Suicidal ideation is prevalent among Chinese adolescents and it is associated with IA and poor QOL. Measures aimed at reducing IA and improving QOL may help prevent suicidal behaviors among Chinese adolescents.

## Introduction

The rapid development of the internet has provided a convenient way for teenagers to acquire knowledge and information, but has also led to a series of psychological and behavioral problems with internet addiction (IA) being one of the most concerned. IA is a mental and behavioral disorder that results from the repeated and excessive use of the internet. It manifests as strong desires to continuously use of the internet, and withdrawal symptoms when use is terminated or reduced. IA is accompanied by mental and physical symptoms, which often co-occur (Shaw & Black, 2008), and results in severe social and psychological impairment (Ko et al., 2012). In China, the age of persons with IA is younger, mainly 15 to 20 years old (Wu, 2013; Bian et al., 2016). Empirical studies have shown that the prevalence of IA among Chinese adolescent students is high, ranging from 10% to 20% (Hu & Wang, 2011; Wang & Meng, 2014; Yang et al., 2010; Yao, 2014; Zhang, Feng & Chen, 2014). IA has become a major mental health issue of contemporary Chinese adolescents.

Adolescence is a vulnerable period of human life cycle, an enormous transition from childhood to adulthood. Many mental health problems emerge during this particularly important period, including depression and IA. It also has been widely accepted that adolescents are at higher risk for non-fatal suicidal behaviors than any other age groups (Liu et al., 2019). Because suicidal ideation (SI) is the most common type of suicidal behaviors and it is a significant risk factor for subsequent attempted and completed suicides (Zhong et al., 2018), many sociological studies focused on modifiable risk factors of SI such as mental health problems and proposed suicide preventions measures based on risk factor findings. In the literature, factors significantly associated with SI in Chinese youths have been reported to be female gender, a low socio-economic status, unhealthy lifestyles, insomnia, personality, and depression (Chao, 2010; Gao, Qu & Miao, 2003; Zhang, Wang & Xing, 2007). In recent years, there has been increasing evidence on the IA-SI association in Chinese youths (Hu & Wang, 2011; Wang & Meng, 2014; Yao, 2014; Zhang, Feng & Chen, 2014). However, because sample sizes of these studies are small and their samples are recruited by using a non-probability sampling technique, the generalizability of existing findings on the IA-SI relationship might be limited. Importantly, most of the studies used samples of university students to investigate the IA-SI relationship. Because nearly all Chinese university students have been adults, who are different from adolescents who are experiencing the transition from childhood to adulthood, it remains unclear that whether the IA–SI relationship also exists in adolescents such as senior high school students.

Findings from a national case-control psychological autopsy study have shown that depression and poor quality of life (QOL) are major risk factors for suicide deaths in Chinese persons aged 15-24 years (Li et al., 2008). A previous study also reported that poor QOL was significantly associated SI among Chinese university students (Chen et al., 2014). Empirical data demonstrate that one-fifth to one-third Chinese middle school students have a QOL below the middle level (Ran et al., 2018). Given that QOL is a broader measure of health that encompasses physical health, role functioning, social functioning, and psychological health (Gu, Xu & Zhong, 2018), poor QOL should also be a strong factor associated with SI in Chinese adolescents. However, empirical evidence for such association in Chinese adolescents is still lacking.

Internet addiction and QOL are two separate but related clinical phenomena. Nevertheless, it remains unclear whether the two variables together are associated with SI, independent from each other, because there is a possibility that, for example, IA would worsen a person’s QOL, which further results in an elevated risk of SI. Investigating the associations of SI with IA and QOL simultaneously would be clinically relevant and inform the development of suicide prevention interventions for adolescents. The present study was carried out to examine relationships between SI and IA and QOL in a large-scale sample of adolescents in western China.

## Materials and Methods

### Subjects

This study was a large cross-sectional survey, which investigated mental health, QOL, and suicidality among senior high school students in Chongqing municipality, located in western China, between March 2013 and November 2014. Chongqing is one of the four municipalities directly under the jurisdiction of the Central Government. This municipality has 38 urban districts and rural counties and a total population of more than 30 million. Multi-stage sampling method was adopted to obtain a representative sample of senior high school students. In detail, 19 districts and counties were randomly selected from the 38 districts and counties during the first stage of the sampling. The second stage randomly selected one or two senior high schools from each selected district or county. Third, all first and second grade students of these selected schools were invited to participate in the current study. Finally, in total, 31,531 students from 29 senior high schools were sampled.

Before the formal survey, the study protocol was approved by Chongqing Health Bureau Ethics Committee. The study procedures were carried out in accordance with the Declaration of Helsinki and related ethical regulations in China. During the fieldwork, all students were told about the purpose of the study, the confidentiality of personal information, and the principle of voluntary participation. Accordingly, students who were willing to participate and signed the informed consent forms were invited to join the study. Parents or legal guardians of students under the age of 16 years were additionally contacted by phone to obtain their informed consent.

### Procedures and assessments

Data were collected with a standardized self-report questionnaire, which contained scales used in this study. This survey questionnaire was administered online. Special measures were adopted to guarantee the confidentiality of students’ personal data. Before the survey, we did a pilot study to test the feasibility of online survey and results from this study showed satisfactory even better psychometric properties of scales administered online than those administered with paper and pencil (Wang, Kuang & Wang, 2012). Details of the special measures to protect privacy and psychometric properties of online scales can be found elsewhere (Wang, Kuang & Wang, 2012).

Demographic variables in the questionnaire were gender, age, and place of family residence (urban vs. rural). Lifestyle factors included smoking and alcohol drinking. Clinical factors included insomnia and depressive symptoms. Three questions asking the numbers of days during the past 30 days on which a student smoked, drank alcohol, and suffered from insomnia. Smoking, drinking, and insomnia were present if the students endorsed at least one day of smoking, drinking, and insomnia. Depressive symptoms were assessed with the depression subscale of the validated Chinese Symptom Checklist-90-R (SCL-90-R) (Liu & Zhang, 2004; Wang, 1984). This subscale has 13 items, containing one item on suicidal thoughts (item 15 of the SCl-90-R: “Thoughts of ending your life”), and the severity of each depressive symptom was rated on a five-point scale (from “0 = no” to “4 = very severe”). To avoid the construct overlap between the outcome of this study, SI and the suicidal item of this depression subscale, item 15 of the SCL-90-R was excluded from the depression subscale. The total score of this subscale varies between 0 and 48, with higher score suggesting more severe depressive symptoms.

Internet addiction was assessed with the validated Chinese Young’s IA Test (Fu et al., 2010). This scale has 20 items, with each being rated on a five-point Likert scale. The total score of the Young’s IA Test ranges between 20 and 100, with a cut-off value of 40, or higher denoting IA. QOL was evaluated with the Chinese Six-item QOL scale (Phillips et al., 2002), which has been widely used in China for assessing the QOL of various populations (Zhong et al., 2019). The six items of the scale assessed six domains of QOL, one item corresponds to one domain, including physical health, psychological health, economic conditions, study, family relationship, and relationships with non-family associates. Each item is rated on a five-point scale (1 = very good, 2 = good, 3 = fair, 4 = poor, 5 = very poor). The total score ranges from 6 to 30, with higher score representing poorer QOL.

In line with prior studies, SI was assessed with the item 15 of SCL-90-R (Li et al., 2014). SI was present if a student rated his/her suicidal thoughts as “mild,” “moderate,” “severe,” or “very severe.”

In this study, the timeframes for assessing depressive symptoms, QOL, and SI were all the month before the survey.

### Statistical analysis

SPSS 20.0 were used for all statistical analyses. Demographic, lifestyle, and clinical characteristic of students with and without SI were compared with independent-samples *t*-test or Chi-square test, as appropriate. The associations of SI with IA and QOL were examined with multiple binary logistic regression that entered SI as the outcome variable, IA and QOL score as two predictors, and demographic, lifestyle, and clinical variables all together as covariates. Odds ratios (95% confidence intervals) were used quantify these associations. All statistically significant levels were set to *P* < 0.05 (two-sided).

## Results

Of the 31,531 sampled students, 27,599 participated in the survey, and 26,688 (84.6%) successfully completed the questionnaire. The average age of the final sample was 16.8 years (standard deviation (SD) = 1.6, range = 15–20 years) and 13,004 (48.7%) were boys. Detailed demographic, lifestyle, and clinical characteristics of the sample are displayed in Table 1.

**Table 1:** Demographic, lifestyle, and clinical characteristics of students from senior high schools in Chongqing, China.

Characteristics	Total sample (*n* = 26,688)	Suicidal ideators (*n* = 3,070)	Non-suicidal ideators (*n* = 23,618)	Statistics	*P*
	*n* (%)	*n* (%)	*n* (%)		
Gender					
Boy	13,004 (48.7)	1,299 (42.3)	11,705 (49.6)		
Girl	13,684 (51.3)	1,771 (57.7)	11,913 (50.4)	χ^2^ = 75.1111	<0.001
Location of family residence					
Urban	9,910 (37.1)	1,189 (38.7)	8,721 (36.9)		
Rural	16,778 (62.9)	1,881 (61.3)	14,897 (63.1)	χ^2^ = 3.789	0.052
Smoking					
No	25,583 (95.9)	2,827 (92.1)	22,756 (96.4)		
Yes	1,105 (4.1)	243 (7.9)	862 (3.6)	χ^2^ = 124.547	<0.001
Alcohol drinking					
No	24,526 (91.9)	2,626 (85.5)	21,900 (92.7)		
Yes	2,162 (8.1)	444 (14.5)	1,718 (7.3)	χ^2^ = 188.574	<0.001
Insomnia					
No	19,289 (72.3)	1,705 (55.5)	17,584 (74.5)		
Yes	7,399 (27.7)	1,365 (44.5)	6,034 (25.5)	χ^2^ = 485.065	<0.001
Internet addiction					
No	19,112 (71.6)	1,547 (50.4)	17,565 (74.4)		
Yes	7,576 (28.4)	1,523 (49.6)	6,053 (25.6)	χ^2^ = 768.534	<0.001
	**Mean (standard deviation)**	**Mean (standard deviation)**	**Mean (standard deviation)**		
Age (years)	16.1 (1.0)	16.1 (1.0)	16.1 (1.0)	*t* = 2.088	0.037
Quality of life score	14.1 (4.0)	17.3 (3.7)	13.7 (3.8)	*t* = 51.509	<0.001
Score of depression subscale of the Symptom Checklist-90-R	18.5 (7.0)	27.9 (9.1)	17.3 (5.6)	*t* = 63.225	<0.001

In total, 3,070 students endorsed SI and 7,576 students had IA. The prevalence rates of SI and IA in senior school students were 11.5% and 28.4%, respectively. The mean QOL score the whole sample was 14.1 (SD = 4.0).

Students with SI had significantly higher QOL scores (17.3 ± 3.7 vs. 13.7 ± 3.8, *P* < 0.001) and higher prevalence of IA (49.6% vs. 25.6%, *P* < 0.001) than those without SI (Table 1). After controlling for demographic, lifestyle, and clinical covariates, IA (OR = 1.15, *P* = 0.003) and a high QOL score (OR = 1.09, *P* < 0.001) remained significantly associated with SI (Table 2).

**Table 2:** Results of multiple logistic regression on associations of suicidal ideation with internet addiction and quality of life, controlling for demographic, lifestyle, and clinical variables.

Variables	OR (95% CI)	*P*
Gender (female vs. male)	1.10 [0.99–1.21]	0.052
Age (years)	0.96 [0.92–1.01]	0.089
Place of hometown (rural vs. urban)	0.97 [0.89–1.07]	0.536
Smoking (yes vs. no)	1.56 [1.29–1.90]	<0.001
Alcohol drinking (yes vs. no)	1.37 [1.18–1.58]	<0.001
Insomnia (yes vs. no)	1.09 [0.99–1.20]	0.068
Score of depression subscale of the Symptom Checklist-90-R	1.16 [1.15–1.17]	<0.001
Internet addiction (yes vs. no)	1.15 [1.05–1.27]	0.003
Quality of life score	1.09 [1.07–1.10]	<0.001

## Discussion

To the best our knowledge, our study is the largest representative cross-sectional survey that investigated the relationships between SI and IA and QOL in Chinese adolescent students. Main findings of this study were (a) 11.5% prevalence of SI, (b) 28.4% prevalence of IA, and (c) significant associations of SI with IA and poor QOL, independent of demographic, lifestyle, and clinical confounders.

Our SI prevalence estimate in students from senior high schools is a little lower than the 13% prevalence in university students (Chen et al., 2010). This prevalence figure also falls within the lower range of SI rates reported by previous studies, for example, in nine countries, prevalence of SI in the general populations was 2.1–18.5% (Weissman, Bland & Canino, 1999), and 4.6–30% of the adolescents reported SI (Lewinsohn, Rohde & Seeley, 1996; Canbaz & Terzi, 2018). Due to differences in SI assessments, timeframes of SI, and populations, results from such direct comparisons might be problematic. Nevertheless, the figure, over 10% of the Chinese adolescents had SI during the recent month, suggests that SI is common among Chinese adolescents. The 28.4% prevalence rate of IA in our sample is similar to those of some previous studies (Fu et al., 2010; Shaw & Black, 2008).

As expected, we found the significantly higher prevalence of SI in students with IA than those without IA, and the significant SI–IA association was independent of depression, QOL, and other clinical factors. This finding is consistent with the elevated risk of suicidal behaviors in Chinese middle school students with IA (Pan et al., 2018), and the positive correlation between severities of IA and SI in Chinese university students (Wang & Meng, 2014; Yang et al., 2010; Yao, 2014). Many possible mechanisms could explain the SI–IA relationship. First of all, IA would lead to poor QOL and depressive symptoms, which in turn result in SI. However, results from our analysis suggest an independent effect of IA on risk of SI, indicating IA may influence risk of SI via other pathways. For example, students may acquire harmful online information about suicide and hold a positive attitude toward suicide, which has been associated with increased risk of SI (Zhong et al., 2018). IA may also increase risk of SI via reducing social contacts, which result in loneliness, a significant risk for SI (Teo et al., 2018).

Our study replicated the significant relationship between poor QOL and SI in adolescent students. Suicide is a complex behavior and many physical, psychological, and social factors involve in the etiology of SI in adolescence (Strandheim et al., 2014). Our measure of QOL included physical, psychological, and social ill-being of an individual, therefore a significant QOL–SI link in adolescent students is expected.

This study has several limitations. First, since this is a cross-sectional study, the causality of the associations between SI and IA and poor QOL can not be determined. Future prospective studies are warranted. Second, because the third grade students were busy preparing for the university entrance exam, no third grade students were included in this study. We must be cautious to generalize our findings to all senior high school students.

## Conclusions

Suicidal ideation is prevalent in Chinese adolescent students and its occurrence is associated with IA and poor QOL. Given the association between SI and attempted and completed suicides, parents, and school teachers of senior high school students should pay more attention to students with SI, and, when necessary, psychiatric assessment and crisis intervention. Because IA is also common in Chinese senior high school students, school-based health education programs aimed at reducing problematic internet use should be useful in suicide prevention. The association between poor QOL and SI suggest that psychosocial supports and other measures for improving QOL of students might also be helpful for preventing suicide.

## Supplemental Information

10.7717/peerj.7357/supp-1 Supplemental Information 1 Dataset.

10.7717/peerj.7357/supp-2 Supplemental Information 2 Variable definitions.

## Additional Information and Declarations

### Data Availability

The following information was supplied regarding data availability: Data is available at Figshare: Zhou, Dongdong (2019): dataset.xlsx. figshare. Dataset. DOI 10.6084/m9.figshare.8293769.v1.
