# Supplementary material for: Internet addiction and poor quality of life are significantly associated with suicidal ideation of senior high school students in Chongqing, China
Source: PeerJ. 2019 Jul 17;7:e7357. doi: 10.7717/peerj.7357 (PMC6719746; doi:10.7717/peerj.7357)
Supplement: Supplemental Information 2 [file peerj-07-7357-s002.docx]

**List of variables in the dataset**

| **Variable name** | **Explanation** | **Type** | **Coding** |
| --- | --- | --- | --- |
| UID | Unique ID |  |  |
| ID | Student number |  |  |
| SI | Suicidal ideation | Category | 1=present; 0=unpresent |
| DEP | Score of depression subscale of SCL-90-R, excluding suicidal item | Continuous |  |
| Gender | Gender | Category | 1=boy; 2=girl |
| Age | Age in years | Continuous |  |
| Place | Place of family residence | Category | 1=urban; 2=rural |
| Smoking | Cigarette smoking | Category | 1=no; 2=yes |
| Drinking | Alcohol drinking | Category | 1=no; 2=yes |
| Insom | Insomnia | Category | 1=no; 2=yes |
| IA | Internet addiction | Category | 1=no; 2=yes |
| QOL | Score of quality of life | Continuous |  |
